# Integrated Analysis of the CircRNA-Based ceRNA Network in Renal Fibrosis Induced by Ischemia Reperfusion Injury

**DOI:** 10.3389/fgene.2021.793182

**Published:** 2022-02-10

**Authors:** Lei Wei, Zhixiang Yu, Limin Liu, Ying Zhou, Xiao Bai, Liya Wang, Ming Bai, Shiren Sun

**Affiliations:** ^1^ Department of Nephrology, Xijing Hospital, Fourth Military Medical University, Xi’an, China; ^2^ State Key Laboratory of Cancer Biology, Fourth Military Medical University, Xi’an, China

**Keywords:** ceRNA network, chronic kidney disease, unilateral ischemia reperfusion injury, RNA-seq, circRNA

## Abstract

**Background:** Circular RNAs (circRNAs), which have broad posttranscriptional regulatory potencies, are involved in the pathogenesis of fibrotic diseases and are promising diagnostic biomarkers and therapeutic targets. However, their specific roles in renal fibrosis remain elusive.

**Methods:** A robust unilateral renal ischemia reperfusion injury (UIRI) mouse model was established to recapitulate the pathophysiology of renal fibrosis. The expression of circRNAs, miRNAs, and mRNAs was profiled by high-throughput RNA sequencing technology.

**Results:** In total, 4983 circRNAs, 216 miRNAs, and 6371 mRNAs were differentially expressed in UIRI-induced fibrotic kidneys. Candidate circRNAs and miRNAs were validated by RT–qPCR in both UIRI and unilateral ureteral obstruction mouse models. Bioinformatic analysis indicated that the parental genes of the differentially expressed circRNAs were predominantly implicated in focal adhesion, adhesion junctions, and regulation of actin cytoskeleton pathways. Through circRNA-miRNA-mRNA construction, we identified two hub genes, circSlc8a1 and circApoe, that targeted a large number of differentially expressed miRNAs and mRNAs related to metabolism and cytokine–cytokine receptor pathways, respectively.

**Conclusion:** CircRNAs were dysregulated in the UIRI model and might be potentially involved in the pathogenesis of renal fibrosis. Research efforts should focus on unravelling the functions of aberrantly expressed circRNAs in renal fibrosis to uncover biomarkers that would enable early diagnosis and the design of prompt therapeutic interventions to prevent disease progression.

## Introduction

Chronic kidney disease (CKD) affects 8–16% of the world population and contributes significantly to global mortality and morbidity (Nelson et al., 2019). Currently, strategies for the early recognition and prevention of the progression of CKD are lacking. Renal ischemia reperfusion injury (IRI) triggered by cardiac surgery, sepsis, and transplantation, among other influencing factors, is not only the most common risk factor for acute kidney injury (AKI) (Ronco et al., 2019) but also the main cause of the AKI to CKD transition (Ó hAinmhire and Humphreys, 2017). Severe IRI primes for the persistent injury and maladaptive repair, manifesting as renal dysfunction, loss of nephrons, microvascular rarefaction, and extracellular matrix deposition, ultimately lead to glomerular and tubulointerstitial fibrosis or renal fibrosis (Shiva et al., 2020). Consequently, further in-depth exploration of the molecular mechanism of IRI-induced renal fibrosis is needed to facilitate the discovery of potential biomarkers and new therapeutic targets for CKD.

Noncoding RNAs (ncRNAs), vital components of epigenetics that make up of more than 90% of the transcriptome, are delicately regulated and play pivotal roles in a range of kidney disorders (Fan et al., 2020; Lin et al., 2020; Chen et al., 2021; Ren and Wang, 2021). Noncoding RNAs are classified into three major categories: microRNAs, long ncRNAs (lncRNAs), and circular RNAs (circRNAs), based on their length and structure. Unlike other ncRNAs, knowledge about circRNAs in kidney diseases is still in the nascent stage. Studies have reported dysregulation of circRNAs in AKI (Xu et al., 2020a), diabetic nephropathy (Bai et al., 2020), immunoglobulin A nephropathy (Luan et al., 2021), autoimmune kidney disease (Zhang et al., 2021), and renal malignancies (Cen et al., 2021). While circRNAs are believed to participate in the fibrotic process (Liu et al., 2019), their specific functions in renal fibrosis remain largely uncharacterized.

CircRNAs are covalently closed loops formed by head-to-tail back-splicing mechanism. They exert their functions through multiple mechanisms, including transcriptional regulation, miRNA sponging, translation into peptides, or interacting with proteins (Zhou et al., 2020). The most explored function of circRNAs is their role as competitive endogenous RNAs (ceRNAs) by sponging miRNAs. A single miRNA can potentially target hundreds or thousands of mRNAs. Thus, the circRNA-miRNA-mRNA network could regulate crucial functions in numerous biological processes, including development, differentiation, proliferation, stress responses, and apoptosis. Moreover, circRNAs are also relatively abundant in tissues and body fluids and highly conserved between species, thereby promoting the translation of findings in rodent models to humans. CircRNAs have a long half-life time and are resistant to exonuclease degradation, therefore holding great promise as ideal biomarkers for disease diagnosis, treatment, and prognosis (Verduci et al., 2021).

In this study, we used the unilateral renal ischemia reperfusion injury (UIRI) and unilateral ureteric obstruction (UUO) mouse models to reproduce the pathological changes of CKD. High-throughput RNA sequencing was performed on kidney tissues obtained from the UIRI mouse model to identify the expression profiles of circular RNAs, miRNAs, and mRNAs. To validate our sequencing results, we performed real-time quantitative PCR of the differentially expressed circRNAs and miRNAs in both the UIRI and UUO mouse models. We then conducted Kyoto Encyclopedia of Genes and Genomes (KEGG) and Gene Ontology (GO) analyses to investigate the potential mechanisms associated with renal fibrosis. Our research based on high-throughput sequencing technology and fundamental experiments broadens the understanding of the role of circRNAs in IRI-induced renal fibrosis and provides a gateway for the development of potential therapeutic interventions for CKD.

## Materials and Methods

### Animal Models

All animal experiments were approved by the Committee on Ethical Animal Care and Use of the Fourth Military Medical University and performed in accordance with the committee guidelines. C57BL/6J male mice were used in this experiment and maintained on a 12-h light and 12-h dark cycle with free access to standard food and water before and after surgery.

### Unilateral Renal Ischemia Reperfusion Model

Three mice as biological replicates were set as either the sham or UIRI group for RNA sequencing. Another five to seven mice who underwent the same surgical procedure comprised the validation group. UIRI surgery was performed as previously described (Jao et al., 2019). Briefly, mice (body weight 20–25 g) were anaesthetized with pentobarbital sodium. Body temperature was maintained at 37°C by a temperature-controlled heating system. A flank incision was made and ischemic injury was induced in the right renal pedicle by nontraumatic vascular clamp for 45 min. The clamp was then removed, and reperfusion of the kidney was visually confirmed by a change in renal color from black purple to pink. One milliliter of saline solution was supplemented, and the flank incisions were sutured in 2 layers. Control animals underwent the same procedure without clamping the right renal pedicle. Mice were euthanized on day 14 after UIRI.

### Unilateral Ureteric Obstruction

UUO, as a canonical renal fibrosis model, was built as the validation group to verify the differentially expressed (DE) circRNAs and miRNAs. Six- to eight-week-old mice (18–20 g) (*n* = 7 in the sham group and 8 in the UUO group) underwent a midline incision. The left ureter was identified and ligated using two 4.0 silk sutures. The midline abdominal incision was sutured in 2 layers. Control mice underwent the same procedure without ligation of the ureter. Mice were euthanized after 14 days post UUO surgery.

### Circular RNA Library Construction and Sequencing

RNA was extracted from kidney tissues using TRIzol reagents (Thermo Fisher, United States). The concentration, quality, and integrity of RNA were determined using the Agilent RNA 6000 nano kit and reagents and the Agilent 2100 Bioanalyzer (Applied Biosystems, Carlsbad, CA, United States), respectively. Standard cDNA libraries were sequenced using the DNBSEQ^®^ platform (BGI-Genomics, BGI-Shenzhen, Shenzhen, China) and 100 bp paired-end (PE100) sequencing was performed on all samples.

### Identification of CircRNAs

Raw reads with adaptors, excessive N content of unknown bases, and low-quality tags were removed using SOAPnuke software (v1.5.6), and the remaining clean reads were used in the subsequent analyses. First, we used Hierarchical Indexing for Spliced Alignment of Transcripts (HISAT2, versionv2.0.4) and Bowtie 2 to map paired-end read to the latest UCSC transcript set (Mus_musculus, GCF_000001635.26_GRCm38.p6). CircRNAs were annotated by computational detection using two separate circRNA algorithms: CIRI and find_circ. Two or more backsplice spanning reads were required to confirm circRNAs in individual samples. The final results are the union of data from the two pipelines. CircRNA expression was normalized using RPB (junction reads per billion mapped reds). DEGseq was used to identify differentially expressed circRNAs. CircRNAs meeting the condition of an adjusted *p* value (for DEG seq < 0.001) and fold change ≥2 were considered to be differentially expressed. When the clustering of junction reads and recording of each circRNA candidate are finished, CIRI scans the SAM alignment one more time to detect additional junction reads. CIRI simultaneously performs further filtering to remove the false-positive candidates generated from the reads incorrectly mapped to the homologous genes or repetitive sequences. The circRNA identification was done by using find-circ software, meeting the following conditions: GU/AG, on the sides of splice site; clear breakpoint; two mismatches; appearance of breakpoint in the position within 2 nucleotides; more than two reads supporting the junction; and a score of blasting to the right position of short sequence that is at least 35 higher than blasting to the other positions. Finally, identified circRNAs were outputted with annotation information.

### Small RNA Library Construction and Sequencing

Small RNAs were isolated from total RNA using PAGE gels by selecting 18–30 nt (14–30 ssRNA Ladder Marker, TAKARA) stripes and recycling. They were purified and ligated to 3 and 5′ adapters. Subsequently, cDNAs were generated by reverse transcription, and PCR amplification was performed to obtain sufficient fragments. The PCR products were purified and tested to ensure the quality of the library prior to sequencing. The expression level of small RNAs was calculated by counting the absolute numbers of molecules using unique molecular identifiers. Differential expression analysis was performed using the DEGseq, and a Q value < 0.001 and an absolute value of |log2 (fold change)| > 1 were set as the default thresholds by which to judge the significance of expression differences.

### Immunoblotting

Frozen kidney tissues were homogenized in ice-cold radioimmunoprecipitation assay buffer (Sigma–Aldrich) containing protease inhibitor cocktails (Millipore). Equal amounts of protein were electrophoresed in 10% sodium dodecyl sulfate–polyacrylamide gel electrophoresis (SDS–PAGE) gels. The resolved proteins were then transferred to polyvinylidene difluoride membranes (Roche, IN, United States). The membranes were blocked in fat-free milk at room temperature and incubated overnight at 4°C with a polyclonal rabbit anti-E-cadherin antibody (1:500 dilution, Bioworld) or a polyclonal rabbit anti-vimentin antibody (1:1,000, Abcam). Blots were washed with Tris-buffered saline and Tween 20 and incubated with horseradish peroxidase-labeled goat anti-rabbit antibody (1:5,000, Sangon Biotech, Shanghai, China) at room temperature for 1 h with gentle shaking. Blots were developed by enhanced chemiluminescence (ECL, Zeta, MA, United States) and visualized with BIO-RAD (BIO-RAD Gel Doc XR+, United States). The protein bands were quantified based on optical densities using ImageJ (version 1.34) and normalized to β-actin.

### Renal Histology

Kidney samples were fixed in 4% paraformaldehyde, embedded in paraffin, and cut into 4-µm-thick sections. The tissue sections were deparaffinized, rehydrated, and stained with hematoxylin and eosin (H&E) and periodic acid-Schiff (PAS) reagents to determine histopathological abnormalities. Collagen was stained with Sirius red, and fibrosis was evaluated by Masson staining. Slides were subsequently scanned by PANNORAMIC 250 (3DHISTECH company, Budapest, Hungary) to examine glomerular, tubular morphology, and collagen deposition.

### Real-Time Quantitative PCR Validation

Total RNA was isolated from kidney tissues using TRIzol reagent. To quantify the amount of circRNA, cDNA was synthesized with the Maxima Reverse Transcriptase (Thermo Scientific, EP0743). RT–qPCR was performed using TB Green® Premix Ex Taq™ II (Takara, Japan) reagent in a LightCycler 480II (Roche Applied Science) according to the manufacturer’s instructions. In particular, divergent primers annealing across the junction sites of circRNAs were used to determine their abundance. The specific primers for circRNAs and miRNAs were designed by Sangon Company (Shanghai), and the sequences were shown in Table 1. The housekeeping gene β-actin was used as an internal control for circRNAs, and U6 was used for miRNAs. The results were calculated using the 2^−ΔΔCq^ method. All experiments were performed in triplicate.

**TABLE 1:** Primer sequences in this study.

Gene name	Primer sequence (5′–3′)	Product length (bp)
β-actin	F: GTG​CTA​TGT​TGC​TCT​AGA​CTT​CG	174
	R: ATG​CCA​CAG​GAT​TCC​ATA​CC	
circOxct1-Divergent	F: GAA​ACT​TCA​ATC​TGC​CAA​TGT​G	98
	R: AAA​TTT​GGT​GTG​GTG​ACG​AGT​A	
circSlc8a1-Divergent	F: TGA​AAC​ATT​GGG​TGG​GAG​AC	112
	R: CTT​TGA​GGA​CAC​CTG​TGG​AGA	
circTinag-Divergent	F: CGA​GGC​GGA​ATT​TGA​ATC​CTT​CT	101
	R: TCG​GAC​ACA​TGC​AGT​TAA​ACT​CA	
circMettl9-Divergent	F: AGG​GTC​ATC​CTG​GCA​TTG​GT	166
	R: CCA​GCC​AGA​TTT​CTC​AAT​GCT​GTT	
circEgf-Divergent	F: GTT​CCA​TCT​GGG​TCA​ATC​CG	100
	R: CAC​AGC​CCA​GGA​CAG​AGG​AC	
circCol1a2-Divergent	F: TAG​CCC​TGG​TGA​ACG​TGG​TG	190
	R: ACC​TCG​GCT​TCC​AAT​AGG​ACC	
circKcnt2-Divergent	F: CAG​CGT​ACA​CAG​TCT​GCA​ATG	124
	R: CCT​GTA​AAC​CCC​ATA​AAG​GTA​GA	
circDcdc2a-Divergent	F: GCA​ATC​CAA​GTC​AAC​CAT​CGG​G	186
	R: CGG​ACA​CGT​TGA​TCC​TGC​TG	
circHavcr1-Divergent	F: TGC​TGC​CAA​TAT​AGA​TGG​TTA​TGC​T	120
	R: AAT​TCA​GGA​GGC​CCC​TCA​CA	
circApoe-Divergent	F: AGGTGTGTGGAGAGCCGC	123
	R: GCTGGAGGAAGTGGGCAA	
Universal U6 Primer F	Sangon Biotech-B661602	
Universal PCR Primer R	Sangon Biotech-B661601	
mmu-miR-142a-5p	GCG​CGC​ATA​AAG​TAG​AAA​GCA​CTA​CT	
mmu-miR-142a-3p	CGC​GCG​TGT​AGT​GTT​TCC​TAC​TTT​ATG	
mmu-miR-135b-5p	CGC​GTA​TGG​CTT​TTC​ATT​CCT​ATG​TGA	
mmu-miR-144-3p	GCG​CGC​GTA​CAG​TAT​AGA​TGA​TGT​ACT	
mmu-miR-107-3p	CAG​CAG​CAT​TGT​ACA​GGG​CTA​TCA	
mmu-miR-192-3p	TGC​TGC​CAA​TTC​CAT​AGG​TCA​CAG	

### Sanger Sequencing

To obtain more reliable PCR results, PCR amplification products were separated by electrophoresis in agarose gels. Briefly, the samples were loaded into precast wells in the gels and then subjected to electrophoresis. Afterward, the PCR products were stained with ethidium bromide, visualized under ultraviolet light, and dissected for subsequent sequencing experiments. For Sanger sequencing, the specific PCR products were extracted and purified directly with the Mag-MK Gel DNA Purification Kit (Sangon Biotech, Shanghai, China) according to the manufacturer’s instructions. The sequences of the products were determined using ABI 3730 DNA sequencers (Applied Biosystems, CA, United States).

### Gene Ontology and Kyoto Encyclopedia of Genes and Genomes Pathway Analysis

GO enrichment analysis was performed on the DE target candidates of DE circRNAs and miRNAs with R software (ClusterProfiler package). KEGG is a database resource for understanding the high-level functions and utilities of biological systems (http://www.genome.jp/kegg/). We used R software for the KEGG enrichment analysis.

### ceRNA Network Construction

MiRanda (http://www.microrna.org/microrna/), RNAhybrid (http://bibiserv. cebitec.uni-bielefeld.de/rnahybrid), and TargetScan (http://www.targetscan.org/) were used to predict the target miRNAs of circRNAs and the target mRNAs of miRNAs. For ceRNA construction, we took the intersection of the target miRNAs identified by the 3 databases above and the differentially expressed miRNAs in our samples. These miRNAs were identified as hub miRNAs. Then we screened miRanda, RNAhybrid, and TargetScan for the targeted mRNAs of the hub miRNAs, and we used the overlap with our sequenced DE mRNAs as the final results. The network was built and visually displayed using Cytoscape software (v3.9.0) on the basis of circRNA--miRNA-mRNA pairs. Different colors represent different expression levels for individual genes.

### Statistical Analysis

All data were obtained from at least three independent experiments and analyzed using SPSS 25.0 (SPSS, Inc., IL) and GraphPad Prism v 8.0 (GraphPad Software, CA). Continuous data are presented as the mean ± standard deviation and were compared using Student’s t tests. A *p* value <0.05 was the significance threshold.

## Results

### Validation of IRI-Induced Renal Fibrosis in Mice

**FIGURE 1:**
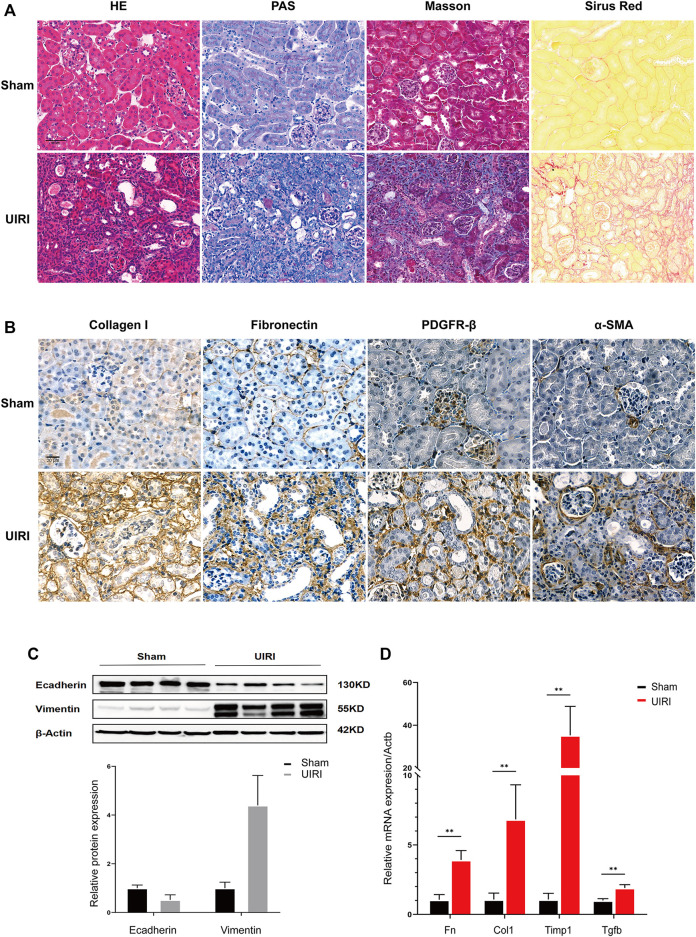
Validation of the transition of UIRI injury to the chronic phase characterized as extensive renal fibrosis. **(A)** Kidney tissues 14 days after IRI displayed tubular necrosis, atrophy, dilation, cast formation, and inflammatory cell infiltration in HE and PAS staining. UIRI kidney tissues showed extensive, severe histological deposition of collagen in kidney tissues in Sirius Red and Masson’s trichrome staining. **(B)** Immunohistological staining showed increased collagen I and fibronectin expression and PDGFR-β+, α-SMA^+^ fibroblasts/myofibroblasts in kidney tissues after UIRI compared to the sham group. Scale bar = 50 µm. **(C)** Representative Western blots showing the change in epithelial marker E-cadherin and mesenchymal marker vimentin protein expression in kidney tissues after UIRI. **(D)** The relative expression levels of fibrotic factors Fn, Col1, Timp1, and Tgfb by the RT–qPCR. **p < 0.01 vs. sham group. Fn, fibronectin; Col1, collagen I; Timp1, tissue inhibitor of metallopoteinase 1; Tgfb, transforming growth factor-beta.

IRI is the injury-specific process triggered by a transient cessation of blood flow, followed by the re-establishment of renal perfusion (Pefanis et al., 2019). Compared to the bilateral IRI (BIRI), UIRI markedly decreases the postoperative death rate, and thus is an ideal animal model to investigate the dynamic pathophysiological mechanism of the AKI to CKD transition and subsequent progression to the end-stage renal disease (Tod et al., 2020). Warm long-term (45 min) ischemia induces a severe injury phenotype of progressive renal parenchymal atrophy and extensive tubulointerstitial fibrosis following incomplete recovery (Fernández et al., 2020; Holderied et al., 2020; Polichnowski et al., 2020). Renal histopathology showed intact structures from control mice who had undergone the sham operation. By contrast, kidney tissues from IRI mice exhibited the extensive renal tubular atrophy, glomerular sclerosis, cast formation, tubule dilation, and inflammatory cell infiltration through HE and PAS staining. Masson and Sirus Red staining revealed excessive extracellular matrix deposition and collagen production (Figure 1A). Immunohistochemistry showed increased expression of the fibrotic markers Collagen I and fibronectin, as well as the fibroblast/myofibroblast markers actin alpha 2, smooth muscle, and platelet-derived growth factor receptor-beta (PDGFR-beta) (Figure 1B). The epithelial marker E-cadherin was decreased, and the mesenchymal marker vimentin was increased in the UIRI group compared to the sham group (Figure 1C). The mRNA levels of fibrotic markers, including transforming growth factor beta 1 (Tgfb1), tissue inhibitor of metalloproteinase (Timp1), fibronectin (Fn), and collagen1a (Col1a), were also elevated significantly after UIRI (Figure 1D). These results indicated that at 2 weeks after UIRI, renal histopathologic examination exhibited signs of extensive tubulointerstitial fibrosis, suggesting that the transition to CKD was already occurring.

### CircRNA Expression Profiles in the UIRI-Induced Renal Fibrosis

**FIGURE 2:**
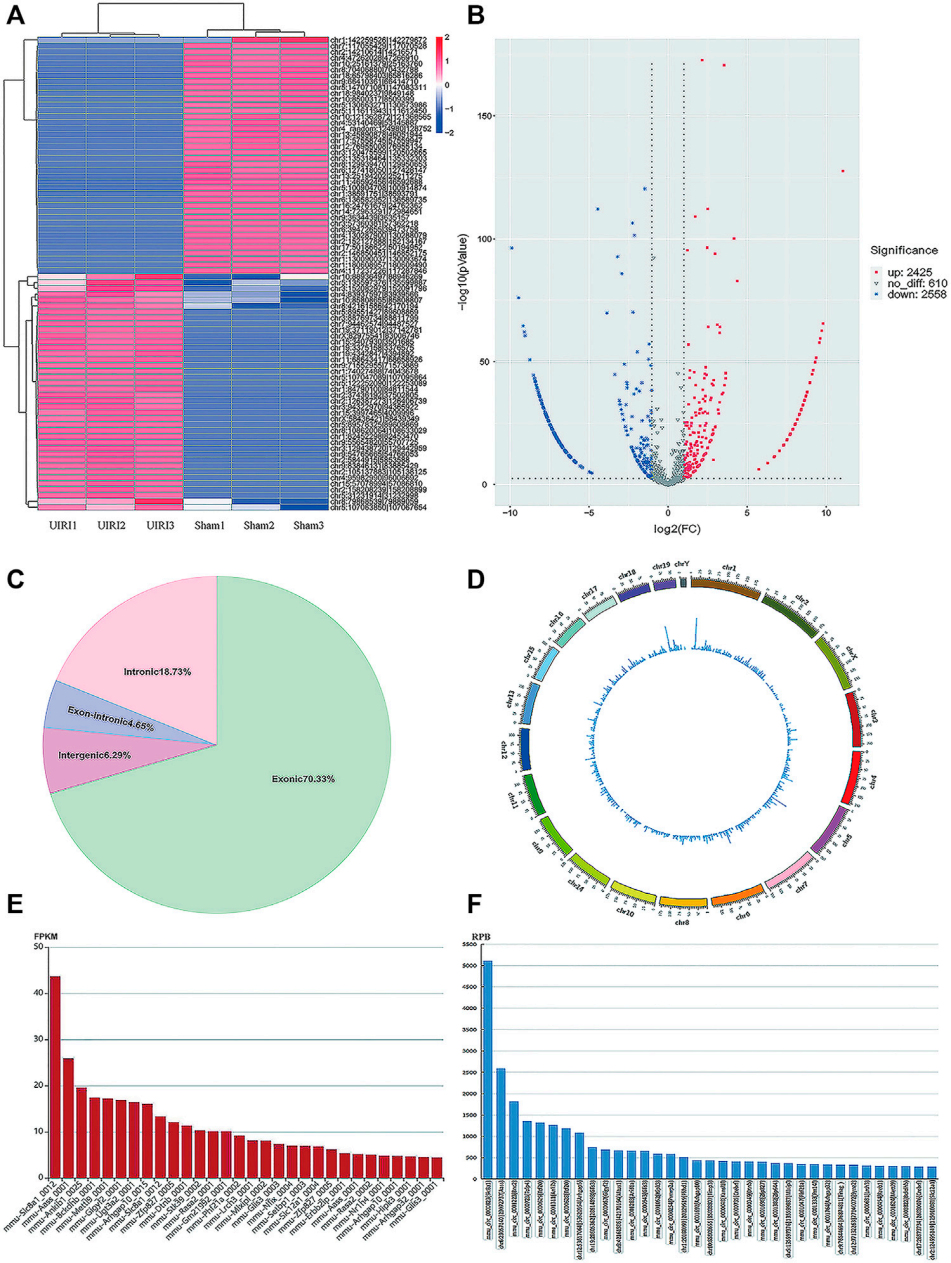
CircRNA expression profiles of the UIRI group. **(A)** The heatmap of representative differentially expressed (DE) circRNAs. **(B)** Volcano plot showing significantly DE circRNAs. Red and blue pixels represent upregulated and downregulated genes, respectively, while gray pixels indicate genes with no significant difference. An absolute value of fold-change > 2.0 and *p* < 0.05 were considered as significantly different. **(C)** The subtypes of the DE circRNAs. **(D)** Circos plot of DE circRNAs illustrating their distribution on the chromosome. **(E)** The top 30 most abundant circRNAs in mouse kidneys from the circAtlas database. **(F)** The 35 most enriched circRNAs in our sequencing data for normal mouse kidneys. **p < 0.01 vs. sham group.

The RNA-seq data analyzed by CIRI and Find_circ software showed that 3409 and 3200 circRNAs were identified in mouse kidney tissues from the sham and UIRI groups (*n* = 3 in each group), respectively. According to the chromosomal location of circRNAs, we concluded that 70.33% of circRNAs were derived from exonic regions and 18.73% were derived from intronic regions, whereas only 6.5% of circRNAs were derived from intergenic regions. Exon-intron (EIcircRNA) and intergenic circRNA accounted for 4.65 and 6.29%, respectively. The Circos plot illustrated their distribution in the chromosomes (Figure 2D). According to a predefined cutoff value, we identified 2425 upregulated and 2558 downregulated circRNAs (log2|fold change|>1, *p* value <0.05) in the UIRI group compared to the sham group (Supplementary Material S1). The heatmap (Figure 2A) and volcano plot (Figure 2B) revealed the DE circRNAs between the sham group and UIRI group. The ten most upregulated and downregulated circRNAs are listed in Table 2. As there is no existing public database of circRNA expression profiles in human CKD or animal models, we compared our sequencing data with the circAtlas database (http://circatlas.biols.ac.cn/), which contains the circRNA expression data of mouse normal kidney tissues. Among the top 30 circRNAs in mouse kidney from circAtlas database, 28 were identified among the 100 most highly expressed circRNAs in our sequencing data (Figures 2E,F). Our data and that of circAtlas suggested that the most enriched circRNA in mouse kidney tissue was circSlc8a1 (mmu_circ_0000823), derived from the exon 2 within the Slc8a1 locus (reference genome NCBI37/mm9).

**TABLE 2:** The top 10 most upregulated and downregulated circRNAs.

Chr:Start|End	Circbase ID	Transcript gene	FC	*p* value	ORF finder prediction
chr13:25194202|25211275	mmu_circ_0004549	Dcdc2a	11.06	2.61E-128	134aa (166->570 nt)
chr2:14210614|14216571	NA	Mrc1	9.80	3.30E-66	NA
chr8:70406880|70432788	mmu_circ_0001682	Psd3	9.72	2.72E-63	165aa (56->553 nt)
chr17:50186622|50194952	mmu_circ_0004549	Rftn1	9.63	3.65E-60	85aa (275–18 nt)
chr18:65798403|65816286	mmu_circ_0000884	Zfp532	9.57	1.70E-58	111aa (4270–3,925 nt)
chr3:120475599|120502665	mmu_circ_0001164	NA	9.37	2.36E-52	148aa (2731–3,177 nt)
chr5:100904708|100914874	mmu_circ_0001368	Lin54	9.31	1.32E-50	316aa (33->983 nt)
chr17:87574135|87708184	NA	Ttc7	9.30	1.98E-50	NA
chr3:135318463|135332303	mmu_circ_0010477	Nfkb1	9.18	2.08E-47	82aa (78->326 nt)
chr1:142259526|142279672	mmu_circ_0000084	Kcnt2	9.18	3.15E-47	None
chr3:129438720|129442959	NA	Egf	−9.90	5.01E-97	NA
chr15:3407930|3501685	mmu_circ_0005598	Ghr	−9.46	4.31E-76	466aa (13213–11813 nt)
chr2:105137883|105138125	NA	Them7	−9.17	1.05E-64	NA
chr1:74027488|74043678	NA	Tns1	−9.10	8.00E-63	NA
chr2:37436192|37502805	mmu_circ_0009934	Strbp	−9.05	2.90E-61	648aa (168–214 nt)
chr2:158208239|158208899	mmu_circ_0001100	Snhg11	−8.74	1.72E-51	80aa (48–290 nt)
chr11:68643417|68658926	mmu_circ_0003258	Ndel1	−8.46	4.89E-44	313aa (13->954 nt)
chr3:88769734|88811799	mmu_circ_0010873	Ash1l	−8.45	9.50E-44	1936aa (101->5911 nt)
chr5:107047089|107095864	mmu_circ_0001380	Zfp644	−8.41	7.01E-43	1292aa (18–3,896 nt)
chr2:5844915|5853588	mmu_circ_0010034	Dhtkd1	−8.41	7.01E-43	131aa (438->833 nt)

Abbreviations: FC, fold change; aa, amino acid.

**FIGURE 3:**
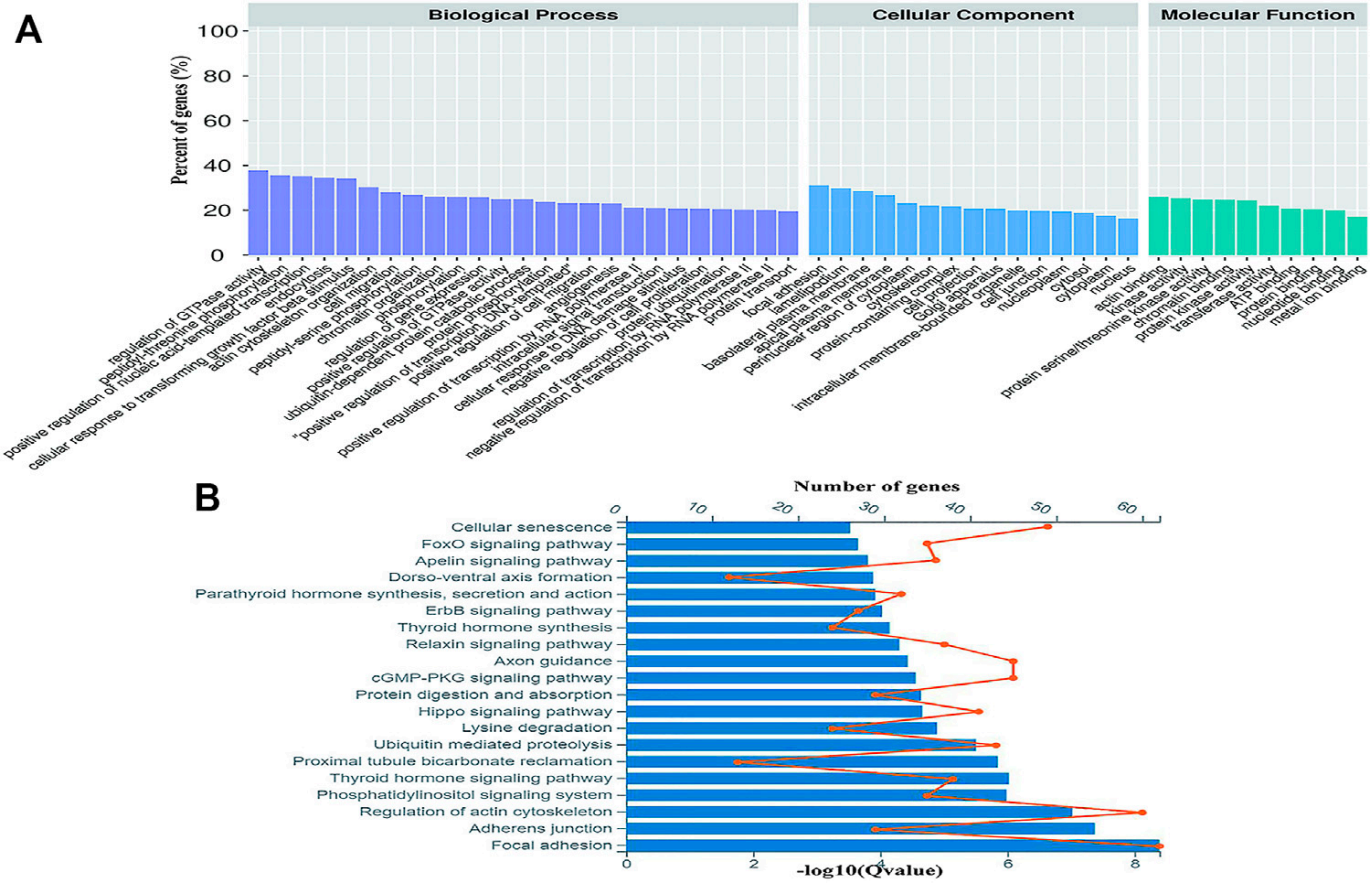
Gene ontology (GO) and Encyclopedia of Genes and Genomes (KEGG) analyses of the parental genes of DE circRNAs. **(A)** Gene ontology analysis on parental genes of the DE circRNAs. The vertical axis shows the annotated functions of the target genes. The horizontal axis shows the number of genes in each cluster. **(B)** Kyoto Encyclopedia of Genes and Genomes analysis of parental genes of the DE circRNAs. The orange node represents the number of the DE circRNAs in each pathway and the value of the blue bars shows the -log10 (Q value).

Gene Ontology (GO) was used to annotate and classify the transcripts of circRNAs based on the GO hierarchical categories of biological process, cellular component, and molecular function. The number of parental genes corresponding to GO entries was determined, and the -log10 (*p* value) was used as the enrichment score. For biological process, the term that contained the most genes was the positive regulation of transcription by RNA polymerase II (GO:0045944, count = 270), and the most significantly enriched term was phosphorylation (GO:0016310, *p* = 2.03E-24). For cellular component and molecular function, the terms that contained the most genes and most enriched were cytoplasm (GO:0005737, count = 1,259, *p* = 5.36E-69) and protein binding (GO:0005515, count = 988, *p* = 2.21E-78) (Figure 3A). The KEGG results indicated that the parental genes of the DE circRNAs were involved in focal adhesion (04510, Gene number = 62, *p* = 4.06E-9), adhesion junctions (04520, Gene number = 22, *p* = 4.22E-8), regulation of actin cytoskeleton (04810, Gene number = 60, *p* = 9.50E-8), thyroid hormone signaling pathway (04919, gene number = 38, *p* = 9.46E-7), and phosphatidylinositol signaling system pathway (04070, gene number = 35, *p* = 1.03E-6).

### miRNA Profiling of UIRI- Induced Renal Fibrosis

**FIGURE 4:**
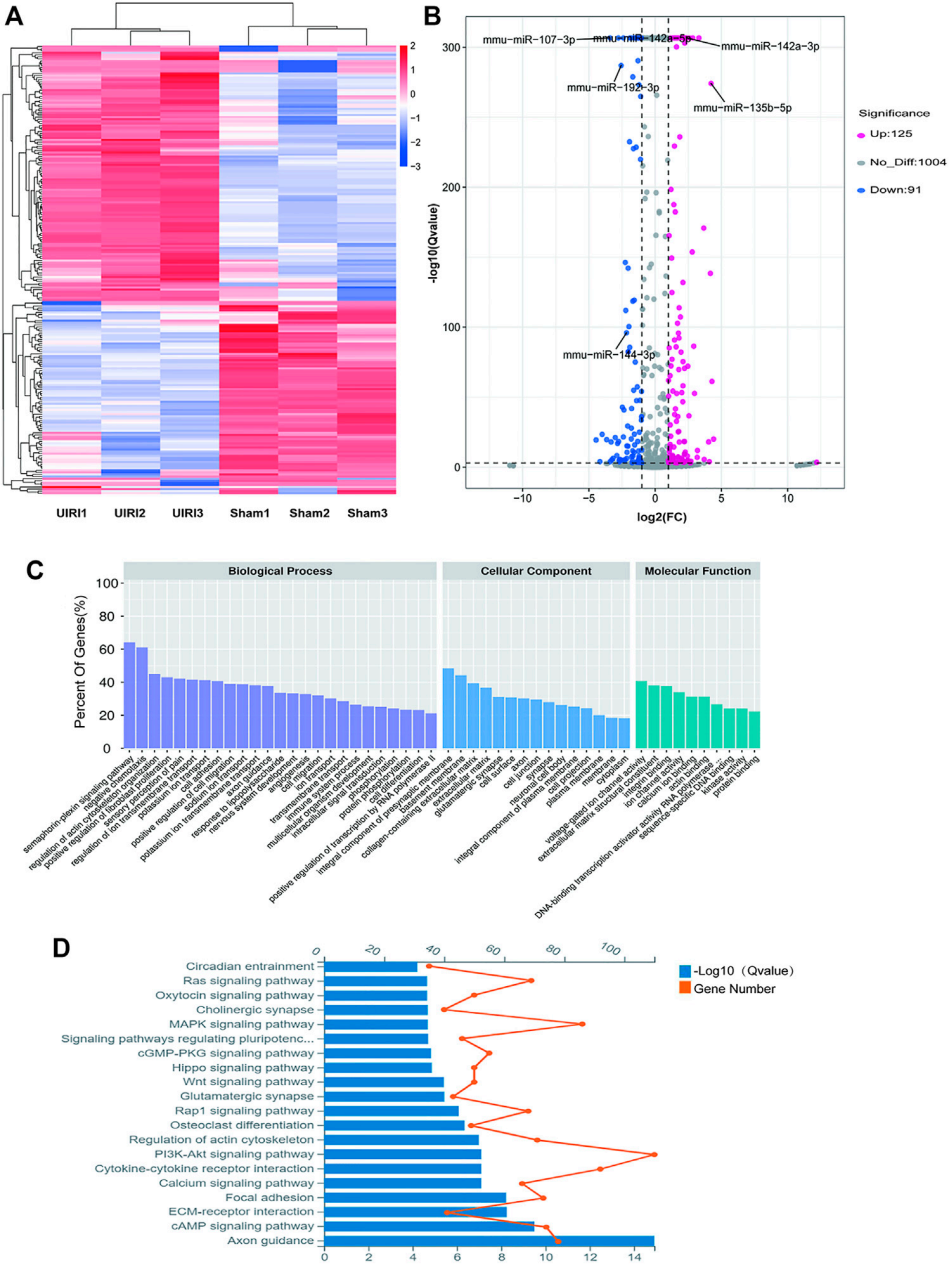
Expression profiles and the enrichment analyses of the DE miRNAs. **(A)** The heatmap of the representative DE miRNAs. **(B)** Volcano plot showing significantly DE miRNAs. Red and blue pixels represent upregulated and downregulated genes, respectively; gray pixels indicate genes with no significant difference. An absolute value of fold-change > 2.0 and *Q value* < 0.001 were considered as significantly different. **(C)** GO analysis of the targets of the DE miRNAs. **(D)** KEGG analysis of the targets of DE miRNAs.

The miRNA expression profiles of the UIRI were also detected. Differentially expressed miRNAs (DEMs) are shown in the hierarchical clustering heatmap (Figure 4A) and volcano plot (Figure 4B). A total of 216 DEMs were identified, among which 125 were upregulated and 91 were downregulated (log2|fold change|>1 and q value < 0.001) (Supplementary Material S2). The 10 most upregulated and downregulated miRNAs are listed in Table 3. To gain insight into the underlying mechanism of IRI-induced renal fibrosis, GO and KEGG analyses were conducted to functionally analyze the target mRNAs of DEMs, which were simultaneously predicted by Miranda, TargetScan, and RNAhybrid and screened by our mRNA sequencing data from the same tissue samples (Supplementary Material S3). The most enriched GO term for the cellular component was plasma membrane (GO:0005886, *p* = 5.46E-40). The most enriched terms for the molecular function and biological process were protein binding (GO:0005515, *p* = 2.87E-56) and cell adhesion (GO:0007155, *p* = 1.74E-54) (Figure 4C). Moreover, the axon guidance pathway (4360) was the most enriched pathway in the KEGG database (Figure 4D).

**TABLE 3:** The top 10 most upregulated and downregulated miRNAs.

Gene ID	log2 (FC)	*p* Value
mmu-miR-135b-5p	4.220048481	5.58E-276
mmu-miR-511-3p	4.167594264	4.03E-140
mmu-miR-147-3p	4.035317806	1.92E-19
mmu-miR-206-3p	3.666410593	1.59E-172
mmu-miR-146b-5p	3.301934805	0
mmu-miR-184-3p	2.971985624	3.62E-54
mmu-miR-31-5p	2.831010469	0
mmu-miR-296-5p	2.798291304	2.02E-155
mmu-miR-31-3p	2.593329329	0
mmu-miR-7043-3p	2.550197083	4.15E-38
mmu-miR-3073b-5p	−4.459775943	8.30E-21
mmu-miR-3073b-3p	−4.000193233	7.51E-25
mmu-miR-107-3p	−3.406435694	0
mmu-miR-1968-3p	−3.17028572	1.21E-07
mmu-miR-7085-3p	−3.011317427	1.46E-22
mmu-miR-1968-5p	−2.834589874	2.07E-18
mmu-miR-874-3p	−2.779516788	0
mmu-miR-551b-3p	−2.760824161	1.02E-19
mmu-miR-3073a-5p	−2.663002978	6.65E-07
mmu-miR-192-3p	−2.567192725	8.63E-289

### Validation of the Candidate CircRNAs and miRNAs by Real-Time Quantitative PCR and Sanger Sequencing

**FIGURE 5:**
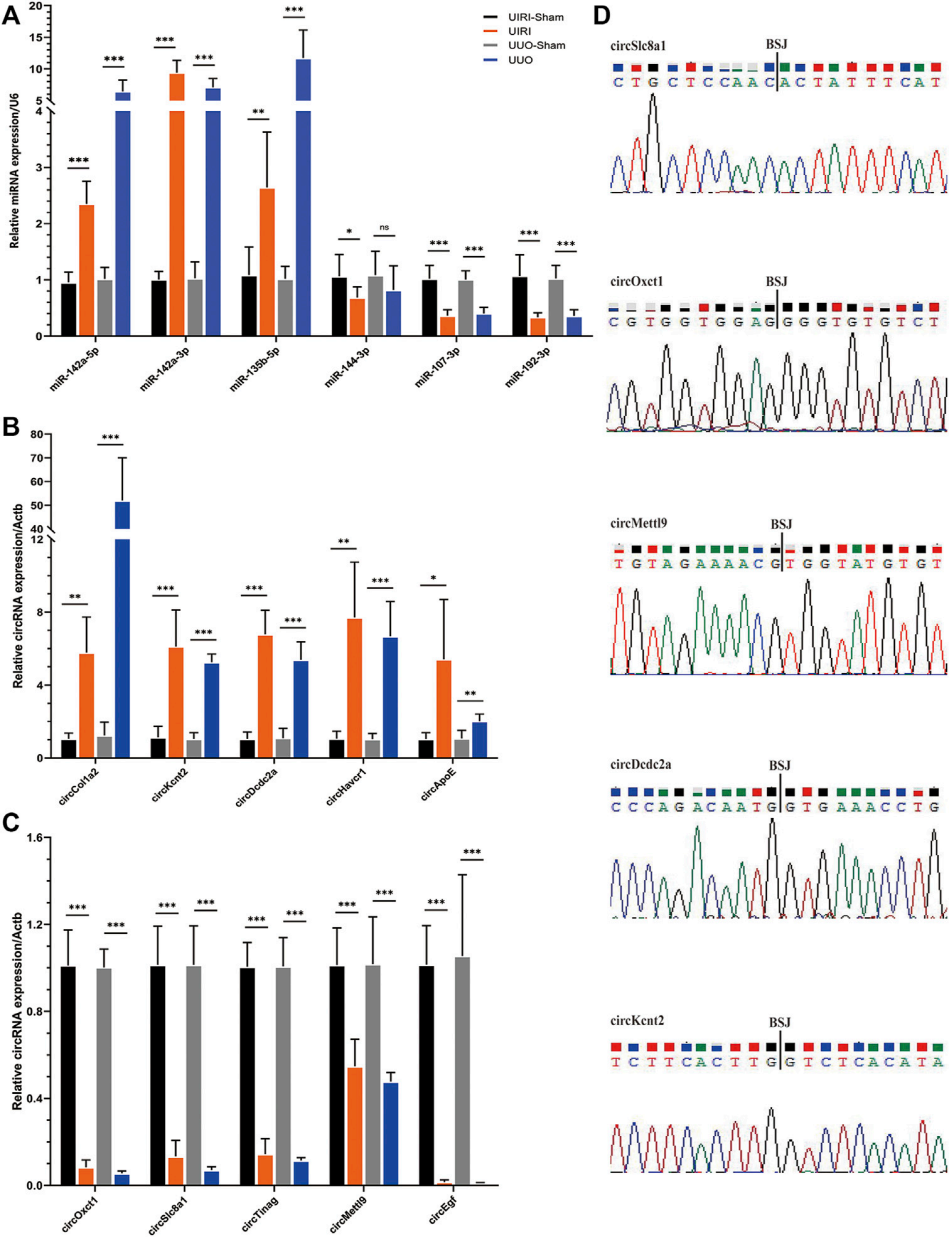
Validation of the circRNA and miRNA expression. **(A)** The relative expression levels of the selected miRNAs detected by the RT–qPCR method. **(B, C)** The results of the five upregulated and downregulated candidate circRNA expression levels by RT–qPCR analysis. *p < 0.05, **p < 0.01, and ***p < 0.001 vs. sham group. **(D)** The back-splicing positions tested by Sanger sequencing. BSJ, back splicing junction.

According to the fold change and expression abundance of the deep sequencing results, upregulated miRNAs (miR-142a-5p, miR-142a-3p, miR-135b-5p) and three downregulated miRNAs (miR-144-3p, miR-107-3p, miR-192-3p) were selected for validation using RT–qPCR in both the UIRI model with an expanded sample size and the UUO model, which is another classic fibrosis model. The RT–qPCR results of the five selected upregulated (circCol1a2, circKcnt2, circDcdc2, circApoe, and circRftn1) and downregulated circRNAs (circSlc8a1, circTinag, circMettl9, and circEgf), were in accordance with those in the RNA sequencing (Figures 5B,C). Sanger sequencing of the PCR products of circRNAs, including circSlc8a1, circOxct1, circMettl9, circDcdc2, and circKcnt2, verified the back splicing junction sites (Figure 5D). Taken together, these results confirmed the accuracy of our sequencing data.

### The CircRNA-miRNA-mRNA Network Construction

**FIGURE 6:**
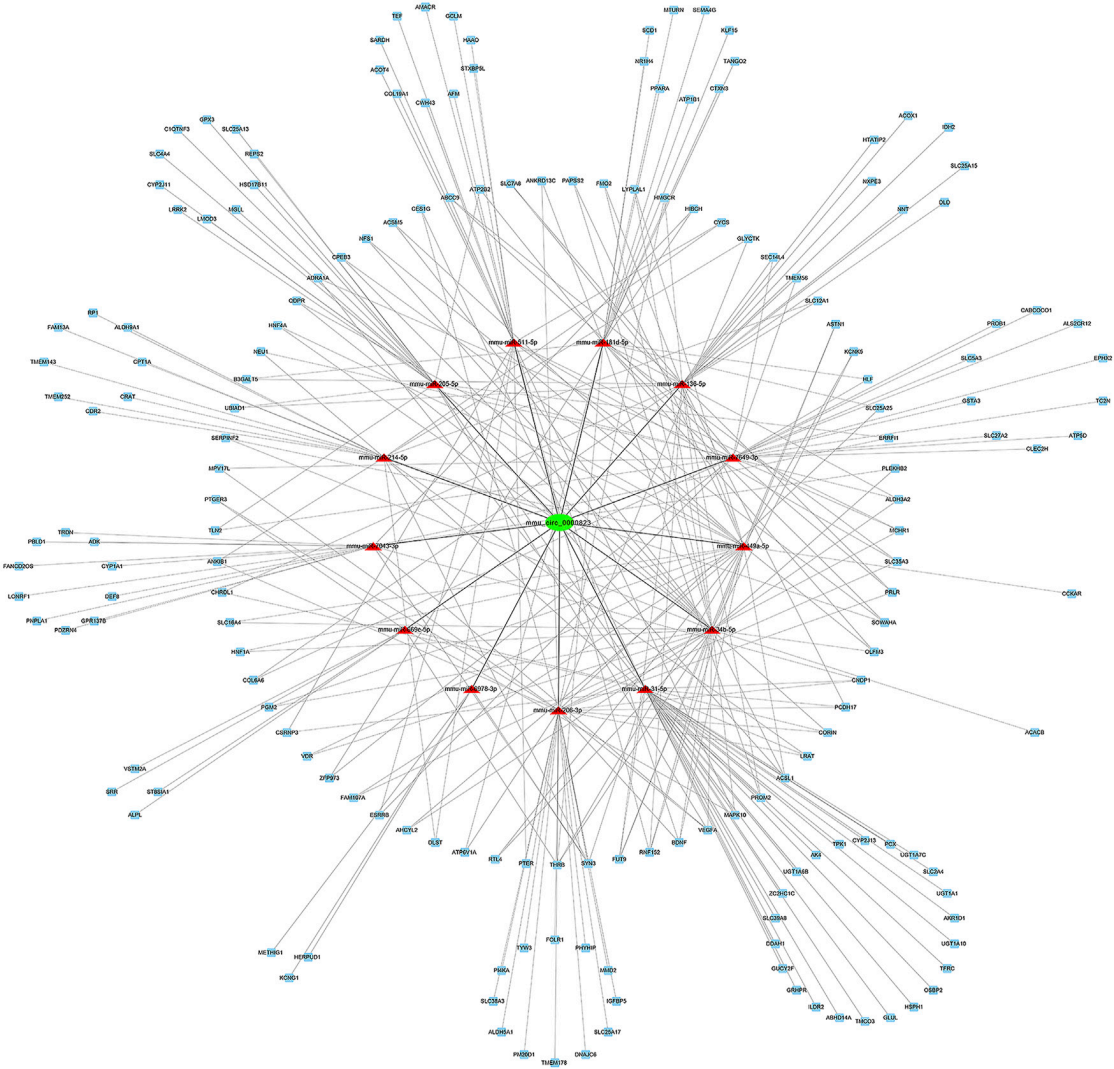
The predicted mmu_circ_0000823 targeted ceRNA network. The colors of green and blue nodes represent the expression levels of the DE genes that were downregulated, while the red nodes represent the upregulated genes. mRNAs in the outer circle can be regulated by a single miRNA, while mRNAs in the inner circle can be targeted by multiple miRNAs.

**FIGURE 7:**
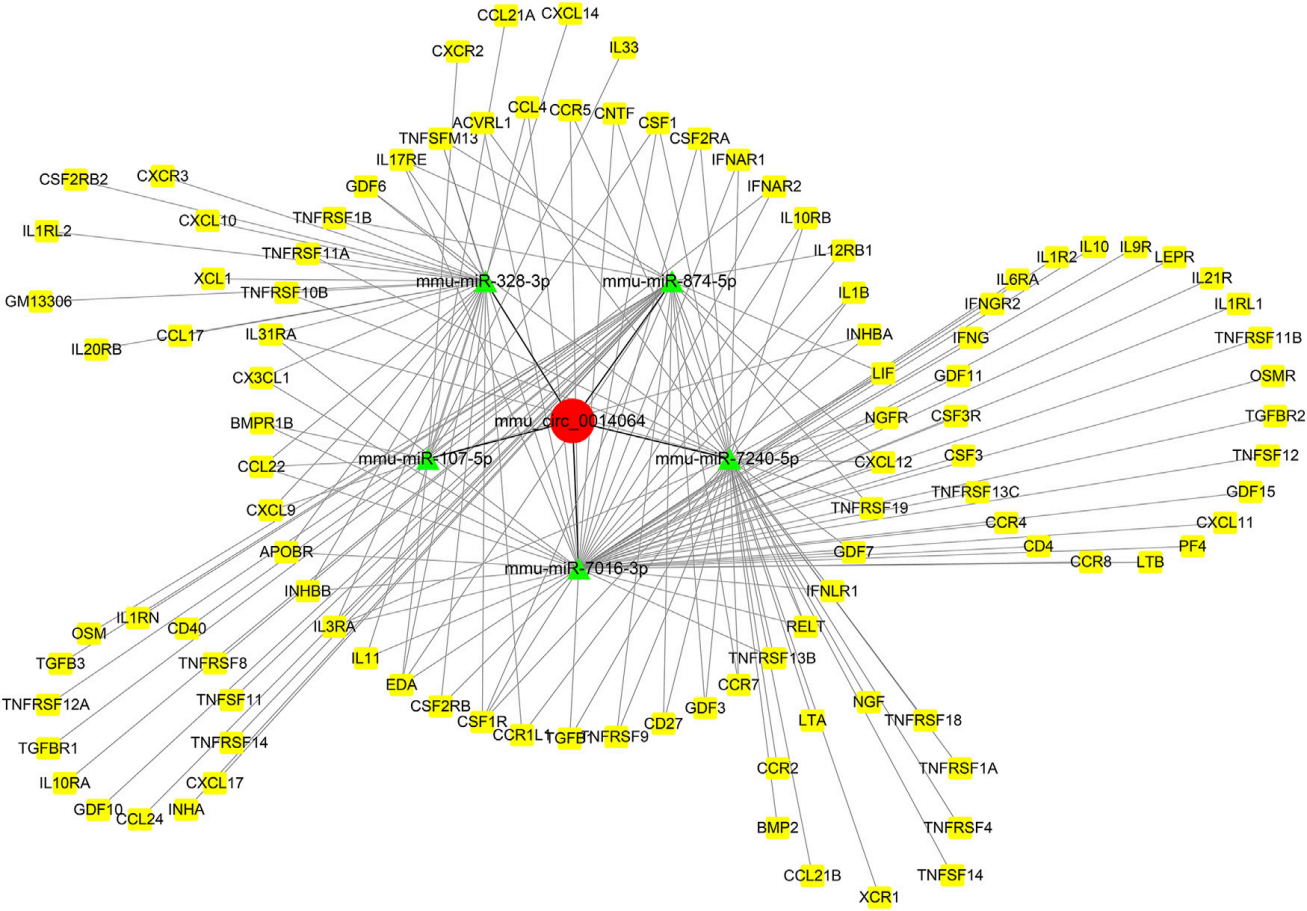
The predicted mmu_circ_0014064-based regulatory network. Green nodes represent downregulated DE miRNAs, while the red and yellow nodes represent upregulated genes.

TargetScan, miRanda, and RNAhybrid were utilized to predict the DEMs targeted by the DE circRNAs and mRNAs targeted by DEMs. In this network, we identified two hub genes, circSlc8a1 (mmu_circ_0000823) and circApoe (mmu_circ_0014064). CircSlc8a1 is the most enriched circRNA in normal mouse renal tissue and was significantly downregulated in the IRI- and UUO-induced renal fibrosis, targeting 13 significantly upregulated miRNAs and the downstream corresponding downregulated mRNAs. The differentially downregulated targeted mRNAs of those 13 miRNAs were mainly enriched in the metabolic pathway, as indicated in Figure 6. CircApoe was significantly upregulated and could sponge 5 significantly downregulated miRNAs, with their target mRNAs enriched in the cytokine–cytokine receptor pathway (Figure 7).

## Discussion

Renal fibrosis is one of the most characterized pathological features of CKD of various etiologies. Progressive fibrosis eventually leads to renal dysfunction, leaving only intensive treatment options of dialysis or transplantation for patients with CKD. Despite the identification of multiple regulatory signaling pathways in past decades, no effective therapeutic strategies have been developed to halt the progression of renal fibrosis. CircRNA is fine-tuned and controlled in many physiological and pathological conditions, and could trigger a significant breakthrough in CKD prevention. Mounting evidence has shown that circRNA expression is altered in kidney disorders (van Zonneveld et al., 2021; Yu et al., 2021). However, the identification and function of circRNAs are largely unknown in the context of renal fibrosis. To address this knowledge gap, we performed systemic transcriptome sequencing of circRNAs, miRNAs, and mRNAs in mouse kidney tissues with UIRI-induced renal fibrosis. We identified a total of 4983 circRNAs, 216 miRNAs, and 6371 mRNAs as differentially expressed. Through comprehensive data mining and bioinformatics analysis, we found two hub circRNAs and their related action network based on our own sequencing data. Our research thus contributes to the body of knowledge that helps us to understand the mechanism of renal fibrosis induced by ischemia reperfusion injury.

Ten candidate circRNAs and six miRNAs were validated by RT–qPCR. The RT–qPCR results showed a consistent expression trend with the sequencing data. The upregulation of miR-142a-5p, miR-142a-3p, and miR-135b-5p, and downregulation of miR-107-3p and miR-192-3p, have been implicated in previous reports as key genes and potential biomarkers in CKD (Jaswani et al., 2017; Gholaminejad et al., 2018; Min et al., 2018; Zhu et al., 2018; Wang et al., 2020). Among the candidate circRNAs, circKcnt2 is highly conserved among species and was reported to regulate the Group 3 innate lymphoid cell functions by inhibiting their activation and resolution of inflammation in colitis (Liu et al., 2020a). However, the primary expressing cell of circKcnt2 in fibrotic kidneys still needs to be clarified. Liu et al. found that the CircOxct1/miR-136/SMAD4 axis inhibited epithelial to mesenchymal transition (EMT) in gastric cancer by antagonizing the TGF-beta pathway (Liu et al., 2020b). Notably, circSlc8a1 (mmu_circ_0000823), highly conserved among different species (*Mus musculus* versus *Homo sapiens*: 88.7% homology), was the most abundant among all of the DE circRNAs and was significantly downregulated in UIRI-induced fibrotic kidneys. CircSLC8A1 was reported to be significantly downregulated in cancers, exerting a tumor suppression function via the ceRNA mechanism. In prostate cancer, circSLC8A1 directly interacted with miR-21 to inhibit the proliferation, angiogenesis, cell migration, and EMT behaviors of tumor cells (Wang et al., 2021). In another study, circSlc8a1 was also significantly reduced in bladder cancer tissues and cell lines. Overexpression of circSLC8A1 inhibited tumor migration, invasion, and proliferation both *in vitro* and *in vivo*. Mechanistically, circSLC8A1 upregulated PTEN by sponging miR-130b/miR-494 (Lu et al., 2019). Interestingly, the downstream targets of circSlc8a1, miR-21, and miR-130b were both dysregulated in our sequencing results and have been reported as biomarkers in CKD (Zhong et al., 2011; Ma et al., 2019). Further bioinformatic analysis of the circSlc8a1-related ceRNA network among the DE miRNAs and mRNAs suggested that it might affect the metabolism of the kidney and be involved in the pathogenesis of renal fibrosis. CircApoe was another hub gene targeting five DE miRNAs and was involved in the cytokine–cytokine receptor pathway. The expression and functions of the rest of the validated circRNAs, including circTinag, circMettl9, circDcdc2a, etc., have been incompletely investigated. Our findings based on bioinformatic predictions and sequencing data provide a more precise scope for subsequent research.

The relationship between aberrantly expressed circRNAs and kidney fibrosis has been debated in several studies. Wen et al. found that circACTR2 was upregulated in glucose-stressed HK-2 cells and promoted renal fibrosis by affecting pyroptosis (Wen et al., 2020). In a UUO mouse model and in TGF-beta-stimulated mouse renal tubular epithelial cells, circRNA_30032 was significantly upregulated and exacerbated renal fibrosis via the miR-96-5p/HBEGF/KRAS signaling pathway (Yi et al., 2021). In a db/db mouse model of type 2 diabetes and renal tubular cells stimulated by high glucose, circEIF4G2 was significantly upregulated (Xu et al., 2020b), while circRNA_010383 was markedly downregulated (Peng et al., 2021). CircEIF4G2 promotes renal fibrosis via the miR-218/SERBP1 pathway. Overexpression of circRNA_010383 inhibited the proteinuria and the accumulation of the extracellular matrix. These findings suggested that circRNAs functioning as ceRNAs by sponging miRNAs are crucial regulators in renal fibrosis. Based on the circRNA-related regulatory network, we identified two hub genes, circSlc8a1 and circApoe, which might be involved in the pathogenesis of renal fibrosis by sponging multiple DE miRNAs and their downstream targeted DE mRNAs. Metabolic disturbance has been increasingly recognized as an important pathogenic process that underlies renal fibrosis, and development of drugs for metabolic reprogramming will be a promising anti-fibrotic therapy (Zhao et al., 2020; Zhu et al., 2021). This study might provide a new perspective on renal fibrosis and contribute to the development of novel therapeutics for CKD.

Our study still has some limitations. Future research should evaluate the quality of our sequencing data by validating more DE circRNAs. The highly conserved DE circRNAs we identified should be examined in kidney tissues from CKD patients to profile their expression levels in the context of renal diseases. The functions of the DE circRNAs need further elucidation, and a direct relationship between circRNAs and their targeted miRNAs should be confirmed. Although the function of circRNAs as miRNA sponges has been the most studied, unearthing other roles, such as encoding peptides or interacting with proteins (Huang et al., 2020; Jarlstad Olesen and S. Kristensen, 2021), will also be critical to deepen our understanding of this new star in the transcriptome.

In summary, the present study offers a systematic perspective on the expression of circRNAs, miRNAs, and mRNAs in IRI-induced renal fibrosis. The results provided convincing evidence that the identified circRNAs and miRNAs are potential biomarkers for renal fibrosis. However, further studies on the target verification and functional analysis of these circRNAs are needed to furnish conclusive evidence to explain the regulatory mechanism of circRNAs in CKD. To better characterize the pathophysiological role of circRNAs, studies should be tailored to (1) further verify circRNA expressions in CKD patients, not only in animal models; (2) delve deeply into the their functions in renal fibrosis by *in vitro* and *in vivo* experiments; (3) explore the cellular molecular mechanism through which the circRNAs exert their functions and try to assess their value as treatment targets; and (4) aim to employ them as diagnostic and prognostic biomarkers in CKD.

Abbreviations; AKI, acute kidney injury; ceRNA, competitive endogenous RNA; circRNA, circular RNA; CKD, chronic kidney disease; DE, differentially expressed; DEMs, differentially-expressed miRNAs; GO, Gene Ontology; IRI, ischemia reperfusion injury; KEGG, Kyoto Encyclopedia of Genes and Genomes; lncRNA, long ncRNA; miRNA, microRNA; ncRNA, noncoding RNA; UIRI, unilateral renal ischemia reperfusion; UUO, unilateral ureteric obstruction.; Ethical Approval and Consent to participate; The animal study was reviewed and approved by the Experimental Animal Ethics Committee of Teaching and Research Support Center at the Fourth Military Medical University, approval number IACUC-2019014.

## Data Availability

The original contributions presented in the study are publicly available in NCBI under accession number PRJNA747937.
