# Supplementary figures and images for: Integrated Analysis of the CircRNA-Based ceRNA Network in Renal Fibrosis Induced by Ischemia Reperfusion Injury
Source: Front Genet. 2022 Feb 10;12:793182. doi: 10.3389/fgene.2021.793182 (PMC8866765; doi:10.3389/fgene.2021.793182)

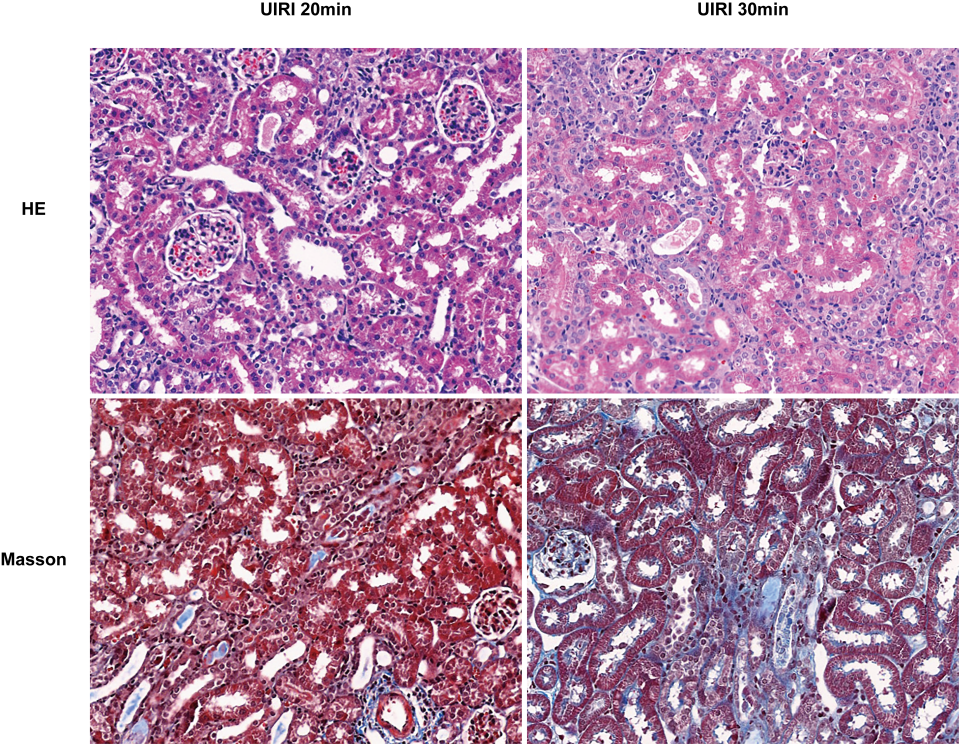


Fig.S2 The HE and Masson staining of kidney injury induced by ischemia lasting 20 min and 30min.

Supplement: Supplementary file 1 [file DataSheet2.docx]
