# Supplementary material for: Integrated Analysis of the CircRNA-Based ceRNA Network in Renal Fibrosis Induced by Ischemia Reperfusion Injury
Source: Front Genet. 2022 Feb 10;12:793182. doi: 10.3389/fgene.2021.793182 (PMC8866765; doi:10.3389/fgene.2021.793182)
Supplement: Supplementary file 2 [file DataSheet1.docx]

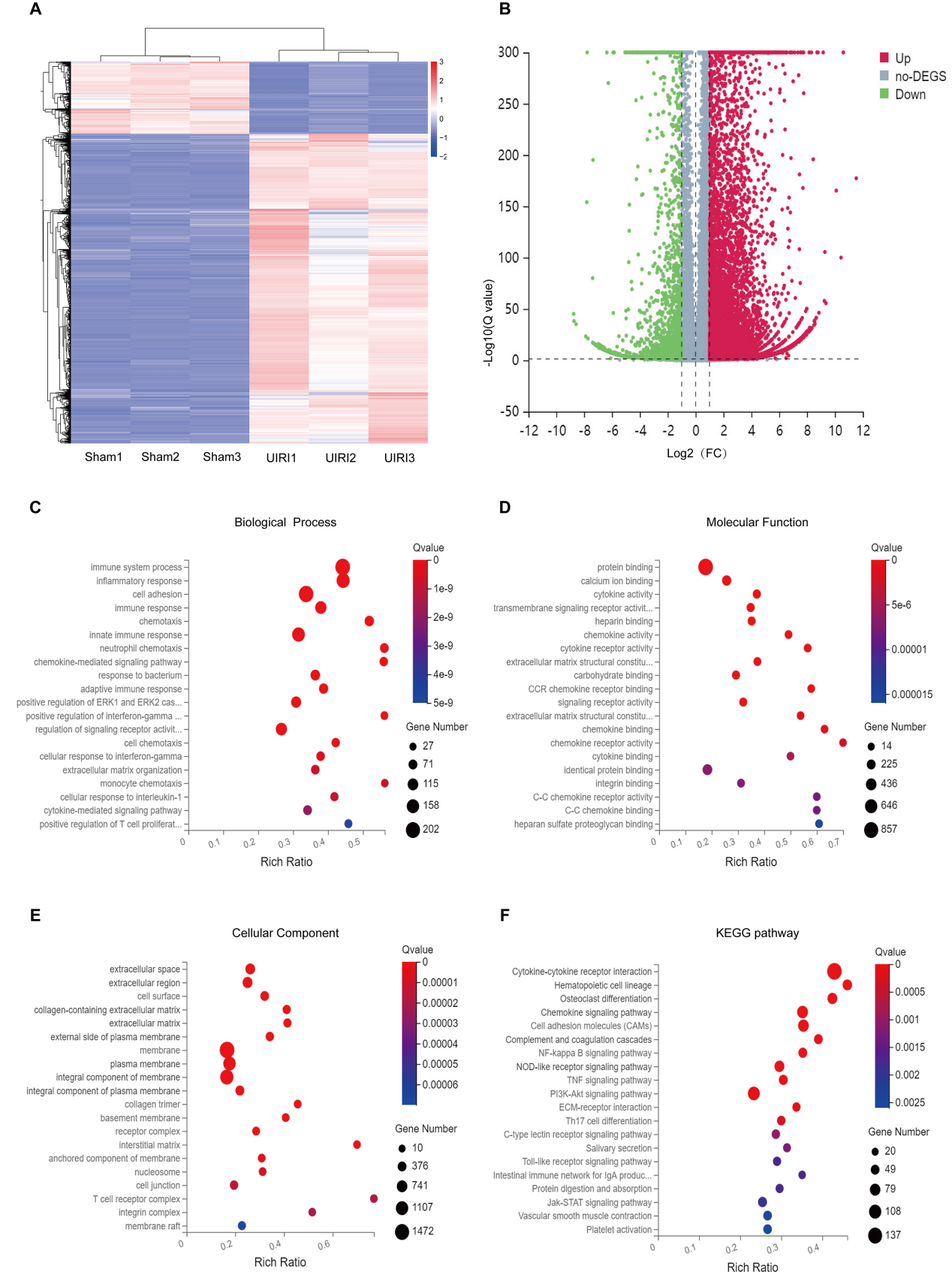


Fig. S1 **mRNA expression profiles of the UIRI group.** (A) The heatmap of representative differentially expressed (DE) mRNAs. (B) Volcano plot showing significantly DE mRNAs. (C) -(E) The 20 most significant biological process (C), molecular function (D), and cellular component (E) terms of GO functional annotation. (F) KEGG analysis of the DE mRNAs.
